# Experimental and Computational Investigation of Biofilm Formation by *Rhodopseudomonas palustris* Growth under Two Metabolic Modes

**DOI:** 10.1371/journal.pone.0129354

**Published:** 2015-06-18

**Authors:** Chase Kernan, Philicia P. Chow, Rebecca J. Christianson, Jean Huang

**Affiliations:** Franklin W. Olin College of Engineering, Needham, Massachusetts, United States of America; LSU Health Sciences Center School of Dentistry, UNITED STATES

## Abstract

We examined biofilms formed by the metabolically versatile bacterium *Rhodopseudomonas palustris* grown via different metabolic modes. *R*. *palustris* was grown in flow cell chambers with identical medium conditions either in the presence or absence of light and oxygen. In the absence of oxygen and the presence of light, *R*. *palustris* grew and formed biofilms photoheterotrophically, and in the presence of oxygen and the absence of light, *R*. *palustris* grew and formed biofilms heterotrophically. We used confocal laser scanning microscopy and image analysis software to quantitatively analyze and compare *R*. *palustris* biofilm formation over time in these two metabolic modes. We describe quantifiable differences in structure between the biofilms formed by the bacterium grown heterotrophically and those grown photoheterotrophically. We developed a computational model to explore ways in which biotic and abiotic parameters could drive the observed biofilm architectures, as well as a random-forest machine-learning algorithm based on structural differences that was able to identify growth conditions from the confocal imaging of the biofilms with 87% accuracy. Insight into the structure of phototrophic biofilms and conditions that influence biofilm formation is relevant for understanding the generation of biofilm structures with different properties, and for optimizing applications with phototrophic bacteria growing in the biofilm state.

## Introduction

Biofilms are characterized by bacterial adherence to surfaces followed by growth to high cell population densities within a self-produced exopolysaccharide matrix. Biofilm formation has the potential to be either problematic or beneficial. In medical contexts, unwanted bacterial growth in the biofilm state can cause disease and exacerbate the development of antibiotic resistance [1,2]. Environmental biofilms can cause biofouling and corrosion in industrial settings that slow productivity [3,4]. Beneficial applications for bacterial biofilms include bioremediation [5], wastewater treatment [6], and biohydrogen production [7,8]. Phototrophic bacteria in particular are important for wastewater remediation and sustainable bioenergy production [9,10,11,12]. A high density of bacteria, such as is achieved in the biofilm state, has been considered desirable to increase efficiency of these nascent technologies [13,14].

There is ongoing interest in understanding the properties, organization, and development of biofilm systems in order to combat biofilm formation when it is problematic, and encourage it when it is desired [15]. Many studies have analyzed factors that influence the formation of heterotrophic biofilms, including the type and availability of nutrients [16,17,18,19], the flow velocities and hydrodynamic effects [20,21,22,23,24], the properties of the attachment surface, and the dependence of the microbial community structures on physiochemical parameters such as oxygen and light [25,26,27]. Several studies have examined factors that influence biofilms formed by mixed species of phototrophic bacteria [28,22,6,29]. This current study of biofilm formation by monospecies phototrophic bacteria contributes to our understanding of how phototrophic biofilms develop in response to light. Knowledge of the dynamics of non-pathogenic strains capable of phototrophic growth in high cell population densities will have important implications for both theoretical research and practical technologies.

*Rhodopseudomonas palustris* is a metabolically versatile phototrophic bacterium that can grow using all four major metabolic modes: photoautotrophic, photoheterotrophic, organoheterotrophic, and chemoautotrophic [30,31]. These bacteria are commonly found in the oxic to anoxic transition zones of natural bodies of water and marsh-like habitats. Under anoxic conditions and in the presence of light, *R*. *palustris* grows via a photoheterotrophic metabolism in which it derives energy from light and can use organic compounds such as succinate or acetate as electron donors. Under oxic conditions and in the absence of light, *R*. *palustris* can grow (organo)heterotrophically, obtaining energy, electrons, and carbon from organic compounds. The versatile metabolism of *R*. *palustris* enables examination of the influences of the metabolic growth mode on biofilm formation within a single species.

In this work, we analyze and compare the biofilms formed by *R*. *palustris* under photoheterotrophic and heterotrophic metabolisms. We experimentally identify differences in their architectures and develop a complementary two-dimensional computational model with the aim of understanding the biotic and abiotic factors that give rise to such differences. The model is a two-dimensional hybrid discrete-continuum model. Hybrid models have been shown to be a good approximation of accurate but more computationally intensive continuum models [32]. Such models describe biofilm colonies as probabilistic cellular automata occupying a discrete grid superimposed on a continuous nutrient concentration field, and have been used by several authors previously to model heterotrophic biofilm development (for a review, see Wang and Zhang [33]). We extended this framework by incorporating light dependence and surface tension effects. The computational model tests the hypothesis that the experimentally observed differences in structure between heterotrophic and photoheterotrophic biofilm growth can be reproduced by considering the dual limitations of diffusive media access, with its effects on porosity, and attenuating light access, with its effects on biofilm depth.

## Materials and Methods

### Growth Conditions

*R*. *palustris* was grown in a defined salts medium [34] with 10 mM sodium succinate as the carbon source. For growth in liquid culture under phototrophic conditions, cultures were grown in deoxygenated medium that was sealed in Balch tubes and cooled after autoclaving with nitrogen gas. Cultures were incubated in front of light from a 60W incandescent light bulb. For growth under non-phototrophic conditions, cultures were grown aerobically in a 30°C incubator that was shaking at 250 rpm. Bacterial biofilms were grown in flow cells using methods adapted from Sternberg and Tolker-Nielson [15]. To initiate biofilm growth, exponentially growing liquid cultures were inoculated into individual biofilm flow cell chambers using sterile syringes with subQ needles (BD, Franklin Lakes, New Jersey). The *R*. *palustris* liquid cultures were inoculated at an optical density of 660 nm between 0.6 and 0.8. One hour after inoculation of the biofilm flow cells, the peristaltic flow of the mineral salts medium was started at a flow rate of 3 ml per minute. Biofilm growth experiments were carried out at 25°C. For photoheterotrophic growth conditions, biofilms were inoculated and grown under anaerobic conditions within an anaerobic chamber (Coy Laboratories, Grass Lake, Michigan) under illumination from a 60W incandescent lightbulb. For non-phototrophic and heterotrophic growth conditions, biofilms were grown aerobically and incubated in the dark with biofilm chambers wrapped in aluminum foil. The biofilms that developed within the flow cell chambers were imaged using confocal microscopy.

### Image acquisition and analysis

Confocal stacks were taken using an inverted Leica SP5 RS AOBS microscope with a 63X glycerol immersion lens. Confocal reflectance at 633 nm was used for cell imaging. For early stage growth, depths of up to 100 μm were easily visible. Anaerobic biofilms older than seven days post inoculation or aerobic biofilms older than ten days post inoculation were found to be too overgrown to provide reliable data due to light attenuation. Attenuation of the light was substantial in these late-stage growth samples with acceptable signal to noise levels obtainable only to depths of 30–40 μm.

Data were collected for six different inoculation dates for both heterotrophic and photoheterotophic biofilm growth conditions with 1–3 replicate samples per inoculation date. Data were collected from 2–14 days post inoculation to observe the full development of the biofilms. On each day that a sample was studied, between 5 and 10 confocal stacks were taken to get a representative sampling of the biofilm structure (see S1 Table). Additionally, a visual survey of the entire sample was conducted to ensure uniformity across the sample chamber. The complete data set includes a total of 324 confocal stacks. Each stack images a 145–160 μm square with depth appropriate to the biofilm thickness to date. Images were spaced at depths intended to optimize for the vertical resolution of the microscope configuration.

For quantitative analysis of these images, we developed software image processing methods based on those provided in the COMSTAT image analysis program created by Heydorn et al. [16]. Whereas the original COMSTAT methods used only one intensity threshold throughout an image stack, our implementation corrects for diminishing light penetration through an adaptive threshold that varies from image to image. These thresholds are selected at specified depths by the user, and then linearly interpolated for all intermediate images. In addition to the analyses present in COMSTAT, we implemented an average area coverage as a function of depth calculation to illustrate the depth-dependent structure of the biofilm.

### Computational Model

We developed a computational model that incorporates abiotic and biotic parameters to describe the growth and maturation of *R*. *palustris* biofilms for the purpose of modeling light-dependent structures observed experimentally. The discrete grid for our model is comprised of uniformly-sized, rectangular cells in the *x*-*z* plane. Each grid cell has two possible states: biomass-filled or media-filled. The grid has finite size (*w*, *d*), with reflective boundaries at the substrate *z* = 0, the bulk media *z* = *d*, and artificial sides *x* = {0, *w*}. For *w* > 10^2^, the difference between periodic and reflective borders becomes negligible; therefore, we used reflective borders to increase computational efficiency.

We considered two separate time scales: (1) nutrient concentration equilibration, and (2) bacterial cell division. Given our experimental setup in which a constant flow of nutrient-rich solution exists above the biofilm, the nutrient concentrations equilibrate instantaneously relative to the much slower process of cell division. We discretized time with two components to each time step:

1. Light intensity and mean equilibrium nutrient concentration are calculated at every grid cell.
2. Biomass grid cells replicate stochastically based on the light and media fields and the existing structure of the biofilm.

In the model, time begins with all grid cells set to be filled with medium except for an inoculation of biomass cells separated equally by an integer distance *s*. Time steps continue until a prescribed biomass count is reached or the probability of division is negligible. Note that we do not consider cell death or structural deformations via hydrodynamic shearing and erosion. In both experiment and model, we are concerned with only early-growth biofilms, rendering cell death a negligible effect. Similarly, flow velocities and structural depths are low enough such that shearing and erosion are rare. Cell motion along the coverslip or within the biofilm is also neglected.

#### Factors affecting cell division

Cell division in the model is governed by access to nutrients in the medium and, for photoheterotrophs, light intensity at appropriate wavelengths. At lower flow velocities, we approximated the fluid dynamics near the biofilm-bulk media interface as forming a boundary layer of velocity-dependent thickness *b* due to large differences in convective and diffusional transfer rates [35]. In two dimensions, we represented this boundary layer using a circular dilation of radius *b*. In bulk media, we assumed that the nutrient concentration is constant, whereas nutrient penetration into the biofilm is achieved solely through diffusion across the boundary layer. We described the concentration gradient using the two-dimensional equilibrium diffusion equation with a reflective boundary at the coverslip and artificial fluid borders:

$$D\nabla^{2}n=u(n)$$ (1)

$$n(\text{bulk media})=1,\frac{\partial n}{\partial z}(\text{coverslip})=0,\frac{\partial n}{\partial x}(\text{border})=0$$ (2)

*n*(*x*, *z*) is the nutrient concentration field measured relative to the bulk media concentration, *D* is the diffusion constant, *u* and is the biological uptake rate for a given concentration. Inside grid cell (*x*, *z*), we follow the approximation made by Hermanowicz [36] and define

$$u_{xz}=u_{0}c_{xz}n(x,z)$$ (3)

Where *u*_0_ is a constant uptake parameter, and *c*_*xz*_ is unity if the cell is biomass-filled and zero otherwise. We simplify (1) by defining a uptake-diffusion ratio *r* = *u*_0_ / *D*, yielding

$$\nabla^{2}n=rc_{xz}n$$ (4)

The uptake-diffusion ratio *r* is related to the Thiele modulus parameters as studied in literature [36]. We find the equilibrium concentrations *n*(*x*, *z*) at each cell using an implicit finite-differences method discretized over the same cellular grid and the UMFPACK unsymmetrical sparse linear system solver [37].

For light-dependent biofilms, the additional factor of the cell division rate due to the light intensity at a given cell was included. We modelled the light with a uniform light source at the coverslip pointing in the $+\hat{z}$ direction. We neglected any backscattered or otherwise-oriented light given its low intensity compared to the vertical light introduced by the incandescent bulb. As follows from the exponential attenuation of light in an absorbing medium, we defined the light intensity at a grid cell (*x*, *z*) to be

$$I_{xz}={\text{I}}_{0}\;\text{exp}\left(-\frac{1}{p}\sum_{i=0}^{z}c_{xi}\right)$$ (5)

where *p* is the characteristic penetration depth of the light.

We translated light intensities and nutrient concentrations into a probability of division per time step via Monod kinetics [35]. We defined the Monod probability factor *K* to be

$$K(y,y_{1/2})=\frac{y}{y+y_{1/2}}$$ (6)

where *y* is a positive dimensional quantity and *y*_1/2_ is the value of *y* at which a cell will divide with probability ½ per time step when all other probability factors are unity. Hence, the division probability of a biomass-filled cell (*x*, *z*) is

$$P{\left(\text{division}\right)}_{xz}=\left\{\begin{array}{c} K(n_{xz},n_{1/2})\;\text{light independent}\\ K(I_{xz},I_{1/2})K(n_{xz},n_{1/2})\;\text{light dependent} \end{array}\right\}$$ (7)

where *I*_1/2_ and *n*_1/2_ are variable model parameters. Note that *I*_0_ is incorporated into *I*_1/2_. Within a single time step, every such *P*_*xz*_ is computed along with a random value *V*_*xz*_, which is drawn uniformly from the range [0, 1]. If *P*_*xz*_ > *V*_*xz*_, then the cell (*x*, *z*) is considered to be dividing.

#### Factors affecting daughter cell placement

The effect of cell division on the morphology of the biofilm is driven by complex biomechanical interactions between parent and daughter cells, as well as their surrounding cell neighborhood. Cell division can create nonlocal perturbations to the morphology through a chain of displacements such that the net effect may be the displacement of an existing cell several cell lengths away from the dividing cell. Since the biomass-filled cells are indistinguishable in our model, the displacement of an existing cell can be modeled as the creation of a daughter cell in its new displaced location (see, for example, Hermanowicz [35]). We approximated such morphological perturbations stochastically under the following assumptions:

- daughter cells must always be connected to the existing biofilm,
- the probability of a perturbation is inversely related to the distance from the parent cell, and
- the probability of a perturbation is also inversely related to changes induced in the biofilm surface energy.

Our model examines a block of *N* -by- *N* grid cells centered on a dividing biomass cell at (*x*_0_, *z*_0_), where *N* is odd and large relative to the perturbation length scale. We immediately assigned a daughter cell placement probability of zero to all biomass cells and to all empty cells not adjacent to a biomass cell, satisfying assumption (a). The remainder of the cells are assigned the probability

$$P{\left(\text{placement}\right)}_{xz}=A\cdot {\left(R_{xz}\right)}^{\alpha }{\left(S_{xz}\right)}^{\beta }$$ (8)

where *R* is the placement distance factor, *S* is the surface energy factor, *α* and *β* are model weightings for the two factors, and *A* is a normalization factor. We define *R* to be the reciprocal of the Euclidian distance:

$$R_{xz}=\frac{1}{{\left(x-x_{0}\right)}^{2}+{\left(z-z_{0}\right)}^{2}}$$ (9)

with the ½ power included in *α*.

While the true surface energy of the biofilm is a function of three-dimensional surface area, we modeled its effect in two-dimensions as a result of changes in the perimeter of biomass cells. Within the *N* -by- *N* block, we considered the perimeter *L* to be the number of grid cell edges that occur between an empty cell and a biomass cell. Then, for a surface tension *γ* and a change in perimeter Δ*L*_*xz*_ produced by a daughter biomass cell at (*x*, *z*), we obtained:

$$\Delta E_{xz}=\gamma \Delta L_{xz}$$ (10)

We translated the energy cost of increased perimeter into the probability factor *S* using Maxwell-Boltzmann statistics:

$$S_{xz}=e^{-\Delta E_{xz}/kT}$$ (11)

Assuming that both *γ* and *T* are constant across the biofilm, we included them in *β* such that:

$$S_{xz}=e^{-\Delta L_{xz}}$$ (12)

Finally, we calculated the normalization factor as:

$$\frac{1}{A}=\sum_{x,z}{\left(R_{xz}\right)}^{\alpha }{\left(S_{xz}\right)}^{\beta }$$ (13)

The *P*(*placement*) may become numerically unstable for very small *R* and *S*. We therefore defined a cutoff value *A*_max_ = 10^12^ above which we do not place a daughter cell. Values of *A* above *A*_max_ corresponded to cells dividing deeply within the biofilm, where the nearest vacant grid cell is on the order of *N* / 2 cells away. Such events are rare as cells are much less likely to divide in the interior of the biofilm. The final daughter cell locations were chosen by iterating randomly though the set of dividing cells and selecting an empty grid cell according to the *P*(*placement*) distribution for the particular dividing cell.

#### Model Parameter Summary

The complete model parameters are shown in Table 1. In summary, these parameters can be directly related to factors influencing the experimental system as follows. The inoculation separation distance is a two-dimensional analog of the initial inoculation density in experiments. The boundary layer thickness is a parameter experimentally related to the flow velocity of the medium past the biofilm during growth with faster velocities resulting in smaller boundary layer thicknesses. The nutrient uptake-diffusion ratio represents the ratio of the rate at which the bacteria consume succinate to the diffusion constant of the succinate through the biofilm. The light penetration depth at the phototrophic wavelength for *R*. *palustris* is a measurable quantity, which we have determined as approximately two microns in late-stage biofilm growth for the experimental system, although based on qualitative observations, this is likely a strong function of biofilm age and density. The Monod parameters, as explained above, are derived from a standard Monod kinetics model of cell division. *α* and *β* are weightings between two approximations of processes involved in the restructuring of the biofilm during cell division, namely the chain of biomechanical displacements caused by the creation of the daughter cell and the presence of a surface tension across the exterior of the biofilm. The mechanical and rheological properties of the biofilm influence the chain of displacements, whereas the nature and composition of the exopolysaccharide matrix influence the biofilm surface tension.

**Table 1:** Parameters and value ranges explored in the computational biofilm model.

Parameter	Symbol	Units	Value(s)
Grid width	*w*	L	128
Grid depth	*d*	L	40
Inoculation separation distance	*s*	L	1–64
Boundary layer thickness	*b*	L	1–16
Nutrient uptake-diffusion ratio	*r*	1/L^2^	0–5
Characteristic light penetration depth	*p*	L	0–30
Light intensity Monod parameter	*I*_1/2_	–	0–1
Nutrient concentration Monod parameter	*n*_1/2_	–	0–1
Cell placement block length	*N*	L	11
Placement distance factor weighting	*α*	–	0–3
Surface energy factor weighting	*β*	1/L	0–3
Placement normalization cutoff	*A*_max_	*L* ^2*α*^	10^12^

While the approximations made within the model prevent exact quantitative mapping of numerical values from experiment to the results from the model, by studying this model we can achieve qualitative understanding of how modification of experimental parameters can be expected to affect the resultant biofilm structure.

#### Random Forest Classifier and Regression Analysis

To evaluate the extent to which the experimentally observed morphology is indicative of the growth conditions, we applied a supervised machine-learning algorithm to analyze data derived from experiment and classify whether a sample belonged to a biofilm formed by photoheterotrophic growth or by heterotrophic growth. Initially, we applied this algorithm to two-dimensional slices of the image stack data. The dataset was comprised of 100 slices extracted from each of the 324 image stacks, yielding a total dataset size of 324,000. Each slice was 96 μm long and 20 μm deep, starting at and perpendicular to the coverslip. For each slice, we provided the machine-learning algorithm with features that were representative of morphology, specifically, the age of the slice in days and 21 samples of the slice’s average coverage vs. depth curve taken every 1 μm from the coverslip up to 20 03BCm deep. Note that the 21 coverage points were normalized to sum to 1 such that they could be compared directly between slices of differing biomass. Each slice in the dataset was labeled with the growth conditions of the biofilm, either 'heterotrophic’ or ‘photoheterotrophic’. The learning algorithm was a random forest classifier with 1000 estimators, a Gini impurity criterion, and a maximum of 5 features considered for each split [38,39].

When applied to results from the computational model, some modifications to the classifier were necessary to make the connection between the model and the experimental slice data, since the model has no inherent physical time scale or space scale. While the number of steps taken by the model to reach a given biomass is analogous to experimental sample age, an accurate conversion is difficult to make; therefore, we used only biomass as a classifier feature and excluded time. For physical scale, assigning each model grid cell to be 1 μm by 1 μm yielded qualitative agreement in feature scale between model and experimental morphologies. We also did not include the first 2μm of coverage data from the model given both the simplifications made when describing the initial biofilm colonization of the coverslip and qualitative differences between the model and experimental results near the base layer. The modified random forest classifier used 19 features: total biomass and the normalized coverage sampled every 1μm from 2 to 20 μm above the coverslip.

The exploration of the model parameter space consisted of 470,000 samples taken uniformly from the parameter ranges described in Table 1. Each sample of the parameter space was grown to a target biomass chosen at random and uniformly from the range of 0 to 40. We used the above classifier to assign a probability of displaying heterotrophic-like morphologies to each parameter space sample. For a coarse evaluation of the effect of model parameters on the assigned heterotrophic probability, we performed a regression analysis using random forests with 500 estimators and a mean squared error criterion [38].

## Results and Discussion

### *R*. *palustris* growth and biofilm formation

**Fig 1:**
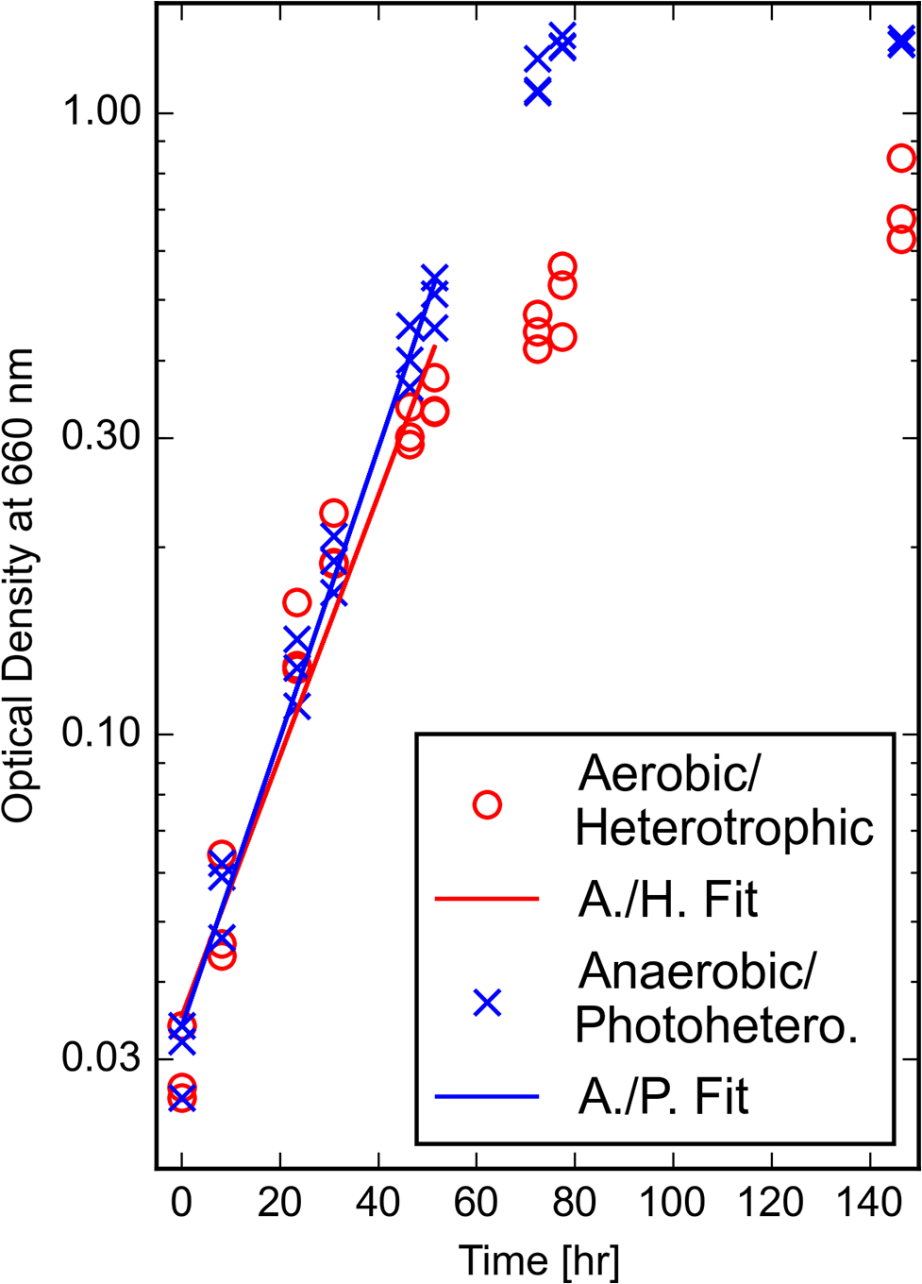
Growth of *R*. *palustris* in batch culture under anaerobic or aerobic conditions. Cultures were grown in triplicate with 10 mM succinate as the sole carbon source. The doubling time of *R*. *palustris* growing anaerobically and phototrophically was 10.8 hrs ± 1.4 hrs, and the doubling time of *R*. *palustris* growing aerobically and heterotrophically was 11.8 hrs ± 1.0 hr.

*R*. *palustris* growing photoheterotrophically in the presence of light in batch liquid culture under anaerobic conditions grew to higher cell yields than *R*. *palustris* growing heterotrophically and aerobically in the dark (**Fig 1**). The doubling time of *R*. *palustris* growing anaerobically was 10.8 ± 1.4 hrs, and the doubling time growing aerobically was 11.8 ± 1 hrs. This is comparable to generation times previously observed in the literature [40]. The yield of the cultures growing phototrophically was 55% greater than the cell yield of *R*. *palustris* growing under heterotrophic and aerobic conditions. The energetic benefit of conversion of light to chemical energy may explain this difference in cell yield.

**Fig 2:**
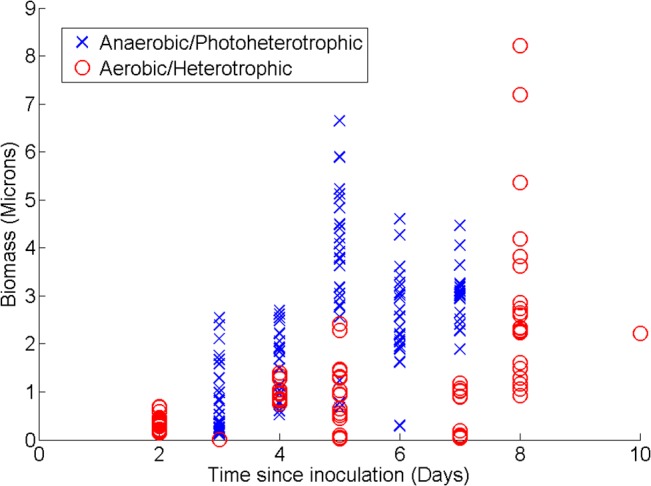
Biomass versus time since inoculation of *R. palustris* biofilms formed under aerobic and anaerobic conditions. A modified implementation of the COMSTAT methods [16] was used to quantify the biomass of anaerobic and aerobic biofilms. Each data point represents an individual scan.

**Fig 3:**
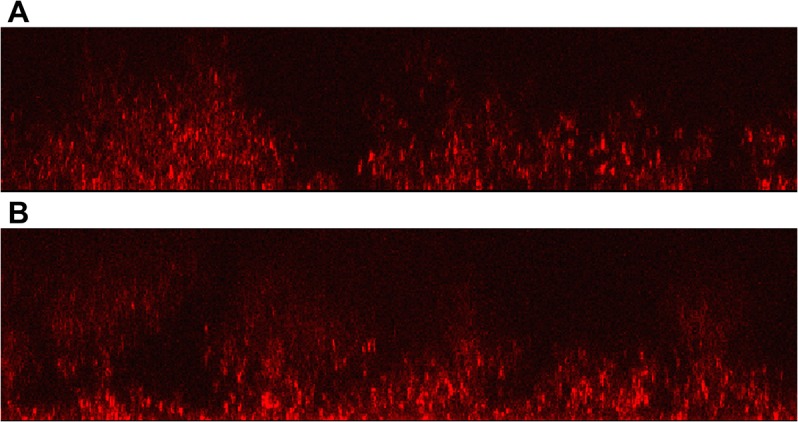
Confocal images of *R*. *palustris* biofilms growing under (A) heterotrophic and (B) photoheterotrophic conditions. Raw vertical images show characteristic morphologies observed in biofilms grown under either of the two growth conditions. Image dimensions are (145 μm x 30 μm) and (145 μm x 35 μm) respectively. The coverslip and light source direction are at the bottom of the images with media above. Biofilms formed under anaerobic and phototrophic conditions show a greater tendency toward ‘T’ or mushroom shaped structures.

*R*. *palustris* biofilms formed under photoheterotrophic and heterotrophic conditions differed in development over time. Similar to growth in batch liquid culture, the biofilms formed by *R*. *palustris* growing anaerobically and phototrophically display a faster increase in biomass than the biofilms formed by the bacterium growing heterotrophically under aerobic conditions (**Fig 2**). During the first five days after inoculation, a difference of means test indicates that the growth rate (biomass over time) of the anaerobic biofilms was significantly faster than the growth rate of the aerobic biofilms (0.44 μm/day vs 0.18 μm/day, *p* < 0.0001). The early stages of biofilm formation of *R*. *palustris* growing via either metabolic mode were characterized by the formation of microcolonies followed by a base layer of cells covering the surface. During later stages of growth under both conditions, the biofilms formed dense, nearly uniform assemblages throughout the depth that could be imaged. However, in the intermediate stages of biofilm formation, clusters of cells formed pillar structures of different and characteristic morphologies depending on whether the bacteria were growing phototrophically or heterotrophically (**Fig 3**). We conducted control experiments to establish if the density of the inoculum could affect the resulting biofilm structure, and we saw no discernable effect over the accessible range of inoculation densities.

Phototrophic *R*. *palustris* biofilms were characterized by pillar-like structures that fanned out at the top into distinctive “T” or mushroom shapes. In contrast, biofilms formed by *R*. *palustris* growing under aerobic and heterotrophic conditions were characterized by pillars that decreased in width with increasing distance from the coverslip. The morphological differences in the biofilms grown via different metabolisms were observed at points of similar biofilm thickness and cell population density indicating that the structural differences observed are not dependent on cell population factors.

**Fig 4:**
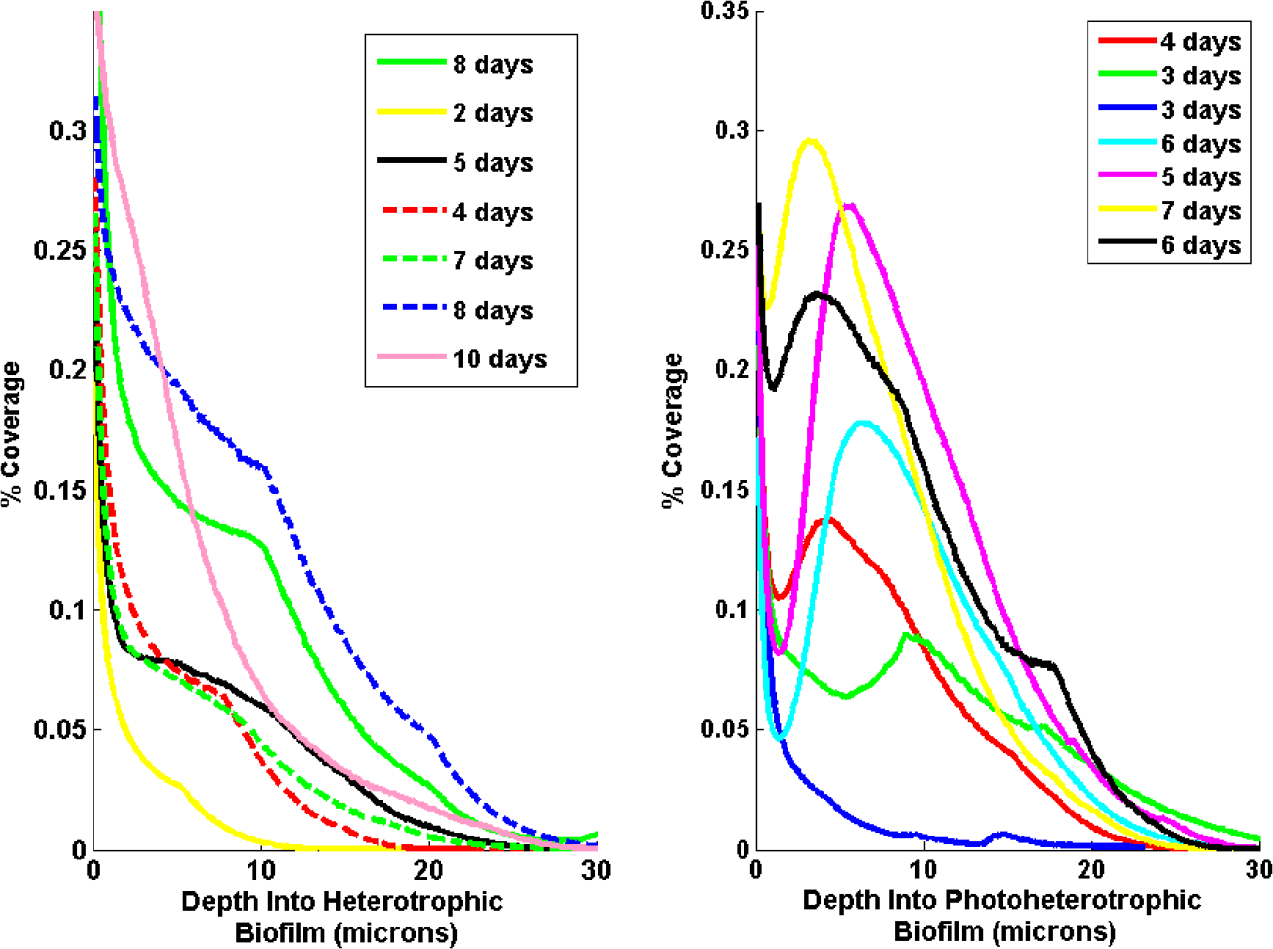
Average biofilm coverage as a function of distance from the coverslip for *R. palustris* biofilms grown under (A) heterotrophic and (B) photoheterotrophic conditions. Each curve is labeled by the time since inoculation and represents an average of multiple scans within each sample obtained by interpolation and binning of values at similar depths. The mean standard deviation for coverage values binned at the same depth was 2.5%. The aerobic and heterotrophically grown biofilms show coverages that decrease monotonically with increasing distance from the coverslip. Very early-growth phototrophic biofilms show similar behavior, but in later stages of growth they show a localized peak in coverage at a depth of 5–10 μm.

We computed the average biofilm area coverage as a function of depth from the coverslip as a representative metric of column shape. The aerobic, heterotrophic biofilms showed coverages that decreased monotonically away from the coverslip at all times after inoculation (**Fig 4A**), while the phototrophic biofilms displayed non-monotonic behavior at intermediate times (**Fig 4B**). For intermediate-time phototrophic biofilms, the coverage is large within the base layer near the coverslip, but, rather than a monotonic decrease in coverage, we see a local maximum in coverage at a distance of 5–10 μm. The qualitative trajectory of the structural development in the biofilm samples is robust to sample-to-sample variations in growth time and local structure (**Fig 4A and 4B**).

The shape of the coverage curve appears to be highly indicative of the growth conditions of the biofilm. To evaluate this, we applied the aforementioned supervised machine-learning algorithm to analyze the experiment and classify whether a sample belonged to a biofilm formed by photoheterotrophic growth or by heterotrophic growth. When applied to the two-dimensional slice dataset, the out-of-bag estimate [38] of the rate of misclassification was 3%. The probability of the supervised classifier achieving a lower error rate when trained on a random permutation of the growth condition labels is much less than 10^−200^. The probability of achieving a lower error rate using a naïve classifier, which guesses the growth conditions based solely on the relative number of heterotrophic and photoheterotrophic slices in the dataset (46% are heterotrophic), is also negligibly small. If we restrict the feature set to only those that are computable from the slice image data by replacing time with biomass as the first feature of the dataset, the out-of-bag error estimate rises to 9%, but the probabilities of that rate or lower for the permuted labels or a naïve classifier remain negligible.

We then applied this classification method to the coverage curves of the three-dimensional image stacks. Using the same depth sampling rate and normalization procedure to produce the coverage features, the trained random forest classifier accurately identified the mode of growth by which the biofilm was formed in 87% of the 324 three-dimensional stacks. A naïve classifier using only the relative number of photoheterotrophic and heterotrophic stacks has a negligible probability of achieving this accuracy. This demonstrates that the morphological profiles of the two growth conditions, as characterized by the coverage curves, are different enough to be recognized with a high degree of accuracy by a machine-learning algorithm.

### Computational Model

We developed the two-dimensional stochastic cellular automaton model described above to examine how abiotic and biotic factors could interact to explain the experimentally observed differences in morphology. We used this model to simulate biofilm development under both heterotrophic and photoheterotrophic conditions. Previous work on modeling biofilm formation has focused on multiparameter, single-species heterotrophic biofilm development [33] with an emphasis on nutrient transport and metabolism with prescribed geometry, sometimes to the detriment of studying morphological development [36]. Continuum and hybrid discrete-continuum models such as ours have had success in reproducing observed biofilm architectures in heterotrophic biofilms [33], but our study of the effects of light sensitivity on the resulting biofilm structure and the comparison of heterotrophic and photoheterotrophic morphologies within a single species is novel. Liao et al. [41] have investigated the effect of optical intensity, but have studied only the effects on model-based optimization of biofilm growth rates and not on biofilm morphology nor in comparison to heterotrophic biofilms of the same species. In addition, our model includes a novel probabilistic method of daughter cell placement incorporating surface tension and distance effects.

Previous work on biofilm models has established a link between media access and the formation of diffuse, fractal-like pillars whose porosity maintains media contact for the largest fraction of cells [36,42,33]. Both the previous work and our model reproduce the early-stage growth observed with both heterotrophic and photoheterotrophic metabolisms in this study. Heterotrophic and photoheterotrophic biofilms require access to the succinate in the media as a source of carbon, but light access is required for growth of photoheterotrophic biofilms. As the photoheterotrophic biofilm grows over time, the intervening biomass between the deep cells and the coverslip attenuates the light available to cells at the deepest levels of the biofilm. Our computational model investigates the structures that result when light effects are included during biofilm growth.

#### Media behavior

**Fig 5:**
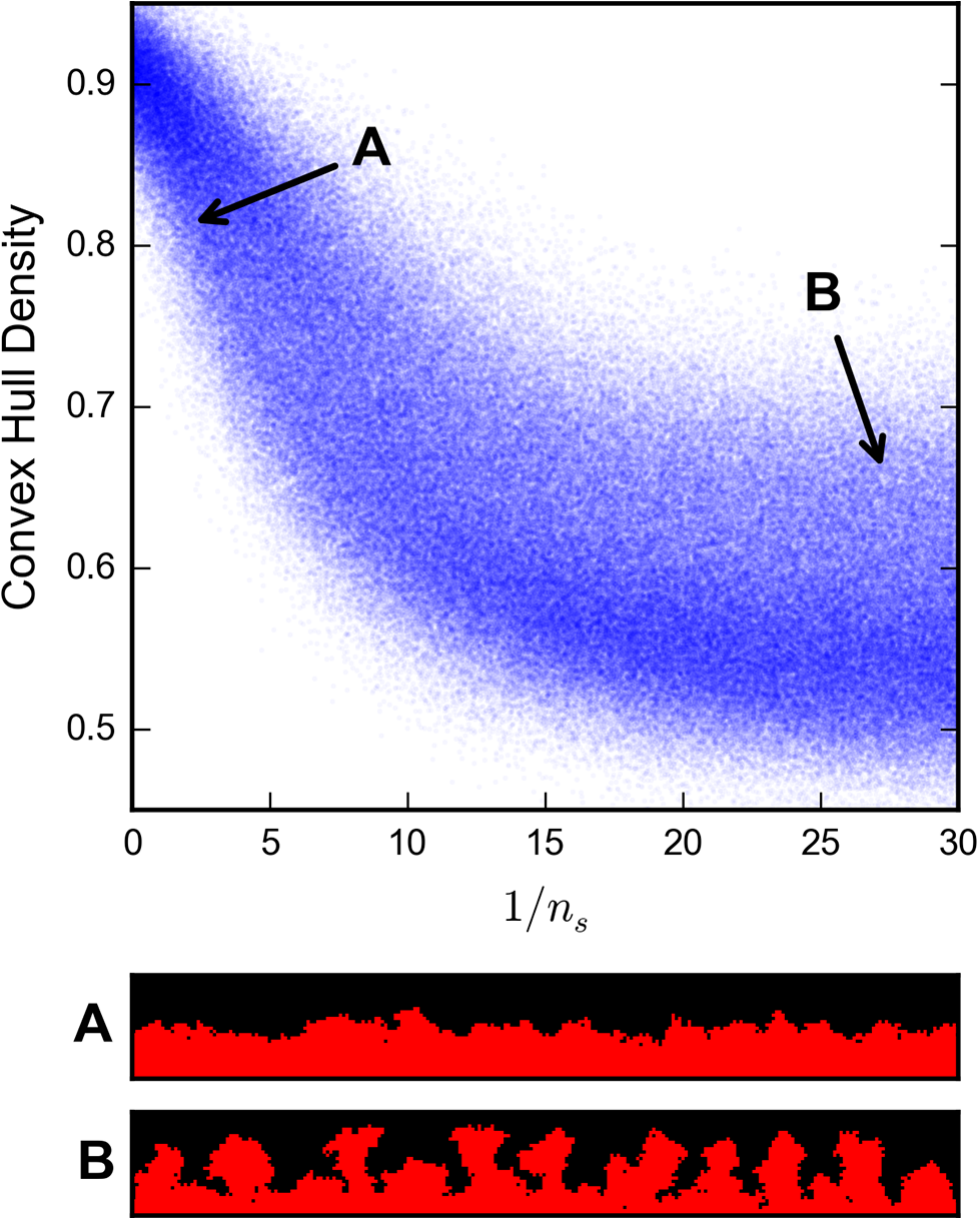
Increasing nutrient scarcity results in increased model biofilm porosity and propensity towards columnar structures. Panel A shows a dense, flat model output produced with *b* = 7 and low nutrient demand *r* = 0.04, while Panel B shows column and mushroom-like growths produced with *b* = 7 and high nutrient demand *r* = 0.56. Nutrient scarcity in the model is characterized by the dimensionless quantity 1 / *n*_*s*_ = *b* ^2^ *r* which is large for nutrient-deprived regimes. We use convex hull density (the density of biofilm-filled cells contained in the convex hull area of the biofilm) as a metric for biofilm porosity. 300,000 samples were taken over the parameter subset (*b*, *r*) such that 1 ≤ *b* ≤ 16, 10^−3^ ≤ *r* ≤ 30, 1 / *b* ^2^ *r* ≤ 30, with fixed values *α* = 2, *β* = 1, and *n*_1/2_ = 0.25. Each of the model samples was grown to the same total mass of 2 × 10^3^ cells.

Our computational model reproduces literature behavior of biofilm morphologies in the absence of light-dependent effects. The Hermanowicz model demonstrated dense, laminar growths for small boundary layer thickness *b*, while increasingly dendritic or columnar growths for large *b* [36]. Our model results in **Fig 5**demonstrate the same behavior and corroborate the more general literature result of irregular columnar growth under nutrient limited regimes that has been previously summarized by Wang and Zhang [33]. Nutrient limited regimes occur in our model when both *b* ^2^ and *r* are large. The parameter *b* ^2^ defines the size of the boundary layer domain in which diffusion dominates over the convective transport in the bulk media and *r* is a measure of the impact of diffusion time on the nutrient uptake rate of cells inside the boundary layer. Small boundary layers denote small diffusion distances and thus small effects resulting from varying *r*, while large boundary layers have large diffusion distances and large effects of *r*.

We characterize the nutrient scarcity resulting from slow diffusion and large boundary layers as the dimensionless quantity *n*_*s*_ = 1 / *b* ^2^ *r*. This quantity is approximately the equilibrium nutrient concentration experienced by an isolated cell as measured relative to the bulk nutrient concentration. Cells on the exterior of the biofilm, which are responsible for most early-stage growth, are at least partially isolated and so *n*_*s*_ is a reasonable measure of the nutrient access on the biofilm exterior. When *n*_*s*_ is small, small differences in distance to the bulk media will result in large differences in nutrient concentrations and thus large differences in growth rates. Hence, any initial fluctuations in cell depth will be exaggerated over time into column-like structures (**Fig 5B**).

#### Light behavior

**Fig 6:**
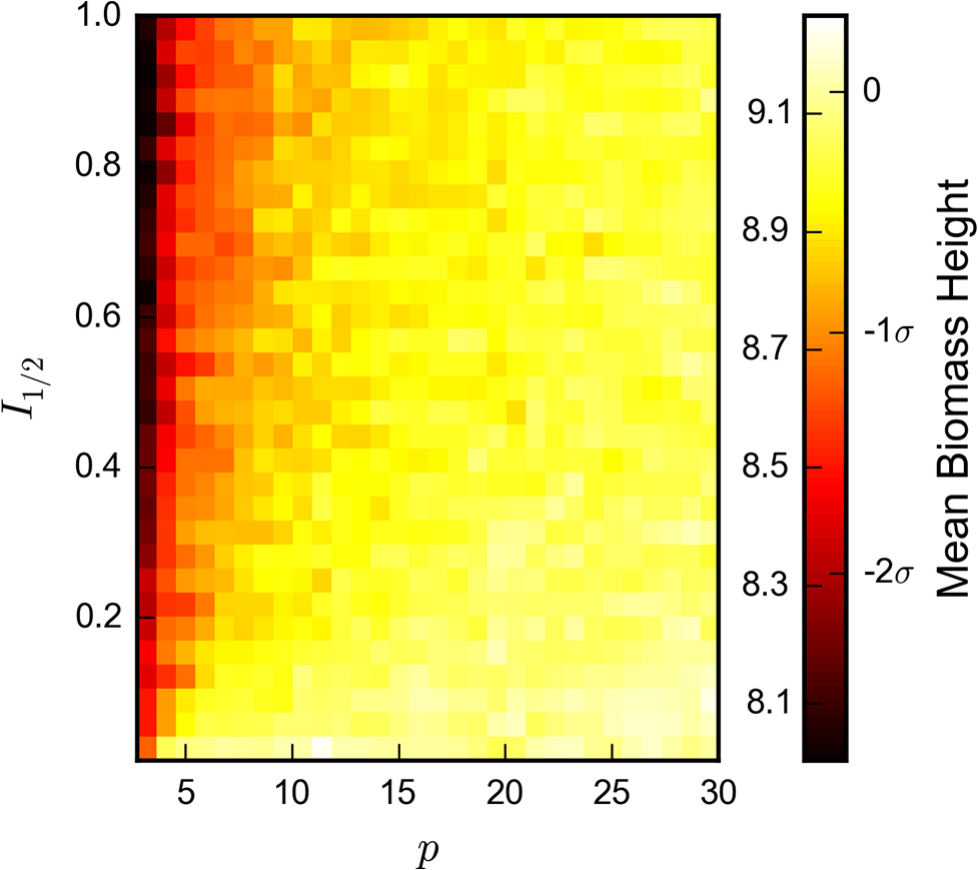
Effects of light-dependence parameters on depth of cell growth. Model biofilms that are light-limited (low *p* and high *I*_1/2_) show decreased average biofilm cell depth. The scale for cell depth is shown with absolute values and in relation to the mean and standard deviation of a population of 10^2^ light-independent samples taken with all other parameters held equal. A total of 96,000 samples were taken with fixed values *α* = 2, *β* = 1, *b* = 7, r = 1/ 2, and *n*_1/2_ = 0.25 Each sample was grown to the same total mass of 2 × 10^3^ cells.

Building upon media-dependent behavior that is consistent with literature results, our model incorporates novel light-dependent reproduction factors. Surveying the broader parameter space, we primarily observe a compression of the biofilm that increases under greater light scarcity and sensitivity. **Fig 6**indicates that when biofilms are sensitive to light intensities (large *I*_1/2_) and light only weakly penetrates biofilm columns (small *p*), the reproduction rates of cells near the top of the column are greatly diminished, which increases the relative reproduction rates of cells closer to the coverslip. This effect is robust and matches experimental data showing deeper average growth for aerobic, light-independent biofilms than anaerobic light-dependent biofilms at the same biomass accumulation.

#### Experimental comparison

In order to evaluate the extent to which, if at all, light dependence in our model induces a transition from columnar to mushroom-like morphologies, we used the modified, biomass-based supervised classifier approach described above to explore the model parameter space. Trained on the experimental two-dimensional slice dataset, the modified classifier achieved a 10% out-of-bag estimate of the rate of misclassification. A naïve classifier would have a negligibly small chance of achieving a lower rate of misclassification. The larger error rate for the modified classifier vs the original classifier reflects the loss of information from excluding the first 2 μm of the coverage vs. depth curve. When applied to the parameter space exploration, the classifier assigned heterotrophic classification probabilities ranging fully from 0 to 1, with a mean of 0.46 and a standard deviation of 0.20. This demonstrates that the model is capable of generating biofilms that have morphologies characteristic of either heterotrophic or photoheterotrophic growth, at least as perceived by the classifier.

We then investigated the relationship of the model parameters to the heterotrophic classification probability using a random forest regression analysis. While the regression only weakly predicted the heterotrophic probability (*R* ^2^ = 0.14), it still provided an estimate of relative model parameter importance in increasing the prediction accuracy (see **Table 2**) [43,39]. The low prediction accuracy could be caused by the wide breadth of morphologies produced by the model across the entire parameter space. The nutrient scarcity and the nutrient concentration Monod parameters had the two highest importance estimates (15% and 14%, respectively), suggesting that the heterotrophic vs. photoheterotrophic morphology production is influenced substantially by nutrient and metabolic factors. The large importance estimate of nutrient scarcity, 1/*n*_*s*_ = *b* ^2^ *r*, in contrast to the small importance of the isolated *b* and *r* parameters also evidences the explanatory power of the dimensionless nutrient scarcity parameter.

**Table 2:** Model parameter feature importances as estimated by a random forest regression over a broad survey of the parameter space.

Parameter	Relative Importance
1/*n*_*s*_	0.151
*n*_1/2_	0.144
*β*	0.143
*I*_1/2_	0.134
*α*	0.134
*s*	0.111
*r*	0.097
*p*	0.075
*b*	0.009

The inoculation separation distance *s* has a non-negligible importance estimate (11%), which matches our observation of its strong influence on produced model morphologies. Experimentally, over the range of accessible inoculation densities, we observed no evidence of a relationship between inoculation density and morphology. It is likely that we are experimentally unable to reach the inoculation densities which the model suggests may strongly influence the biofilm morphology, but it is also possible that the large variance in morphological classification induced by the separation distance is further evidence of the model inaccuracy near the coverslip.

Most importantly, the light penetration depth (8% estimated importance) and light Monod (13%) parameters do not dominate the regression of heterotrophic classification probability. The nutrient scarcity, nutrient concentration Monod, and daughter cell placement parameters each had greater or equal importances than the light Monod parameter (see **Table 2**). This suggests that light dependence is not required in the model to induce a transition from columnar to mushroom morphologies. However, there may yet be some parameter subspace where light drives the morphological transition as the importance estimates derived from the regression do not capture the complex relationships between parameters.

**Fig 7:**
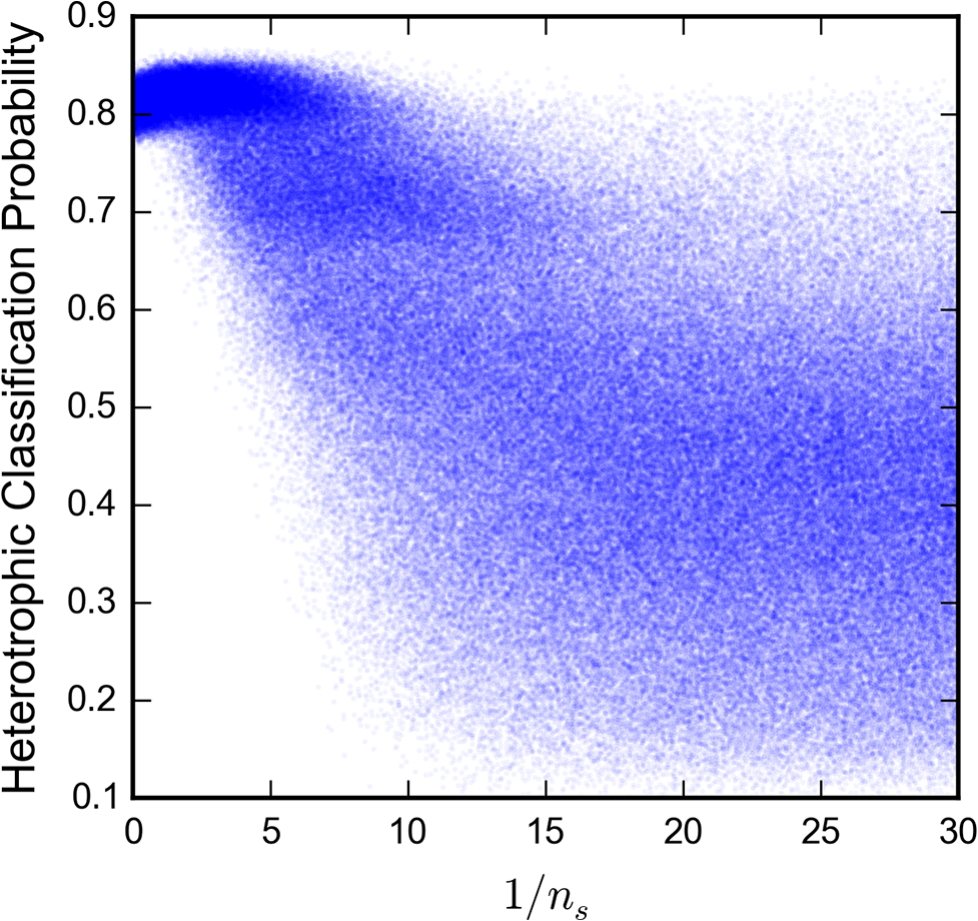
Increasing nutrient scarcity results in decreased heterotrophic classification probability of model output. The random forest classifier was trained on the 324,000 slice experimental dataset with 20 features: biomass and coverage sampled every 1μm from 2 to 20 μm above the coverslip. The classifier was applied to the same 300,000 samples of the model parameter space described in **Fig 5**. Classification probabilities closer to 1 indicate a model output that more closely resembles the heterotrophic experimental slices than those closer to 0, which resemble photoheterotrophic slices. The shape of the relationship between 1/*n*_*s*_ and heterotrophic classification probability generally matches that of 1/*n*_*s*_ and convex density, as presented in **Fig 5**.

To further investigate the influence of nutrient and light parameters, we examined a parameter subset that still produced both heterotrophic and photoheterotrophic morphologies with all other parameters held constant. In **Fig 7**, we show that varying *n*_*s*_ with no light dependence has a clear influence on the heterotrophic classification probability, which closely resembles the influence on convex hull density in **Fig 5**. Nutrient scarcity appears to promote mushroom structure formation and produce photoheterotrophic-like morphologies, while its abundance produces heterotrophic-like morphologies. For light penetration depths high enough (p > 3) to permit complex morphologies to form, varying either of the light parameters does not substantially affect the distribution of aerobic classification probabilities. Hence, based on the model, the transition from photoheterotrophic to heterotrophic morphologies can be induced by a change in nutrient parameters alone.

The computational model therefore suggests that the morphological differences seen experimentally between heterotrophic and photoheterotrophic biofilms may not be solely a result of a direct response to the availability of needed light, but may also be the result of other factors including surface tension and changes in chemical nutrient demand and sensitivity. While the surface area reducing effect of increased surface tension is a clear link to biofilm morphology, we currently have no evidence of differences between the exopolysaccharide of photoheterotrophically and heterotrophically grown biofilms that could result in this change in surface tension. Rheological measurements of biofilms grown in the two different states could establish whether this is a factor. Changes in chemical nutrient demand and sensitivity could clearly arise from the differences in metabolism and energy source utilization between photoheterotrophically and heterotrophically grown biofilms, but the origin of the link between these model parameters and the resulting morphology is less clear. In practice, future experiments using different nutrient sources during biofilm growth such as compounds with different diffusivities and/or which stimulate different metabolic activity by the bacteria could help to clarify these potential influences. In summary, the tendency of light-dependent biofilms to form mushroom structures may not be solely explained by the abiotic light level gradient across the biofilm. Additional biotic factors are likely needed to explain the morphologies observed.

## Conclusions

We find that *R*. *palustris* growing via photoheterotrophic or heterotrophic metabolisms develop biofilms with different architectures. We analyzed confocal laser scanning microscopy image data from replicate *R*. *palustris* biofilms grown via different metabolic modes and collected quantitative data on biofilm structural parameters for each data set. These data indicate substantial differences in architecture between biofilms formed under different metabolic modes, as characterized by biomass coverage as a function of distance from the coverslip. We interpret these differences in biofilm structure as evidence of the different pressures on the growth and development of populations of phototrophic bacteria that result from the use of light for energy.

The two dimensional cellular automata computational model that we develop suggests that the light gradient across the biofilm alone may have a smaller influence than other factors, like metabolic nutrient demand, on the development of the observed structures. This understanding of the effect of metabolism on biofilm development contributes to an understanding of biofilm formation for all biofilm systems, while also providing insight into the optimal growth conditions for applications that involve environmental biofilms.

## Supporting Information

S1 Table Information on Experimental Data Collection. This table shows a comprehensive listing of all the experimental data including sample and scan replicates analyzed for this work.
(DOCX)
