# Supplementary material for: Experimental and Computational Investigation of Biofilm Formation by Rhodopseudomonas palustris Growth under Two Metabolic Modes
Source: PLoS One. 2015 Jun 18;10(6):e0129354. doi: 10.1371/journal.pone.0129354 (PMC4472842; doi:10.1371/journal.pone.0129354)
Supplement: S1 Table — This table shows a comprehensive listing of all the experimental data including sample and scan replicates analyzed for this work. (DOCX) [file pone.0129354.s001.docx]

**Table S1.** Information on experimental data collection.

| Growth Condition | Inoculation Date | Data Collection Date | Days Since Inoculation | Number of Samples | Number of Scans |
| --- | --- | --- | --- | --- | --- |
| Aerobic | 25-Feb | 28-Feb | 3 | 1 | 2 |
| Aerobic | 25-Feb | 2-Mar | 5 | 2 | 6 |
| Aerobic | 25-Feb | 4-Mar | 7 | 2 | 6 |
| Aerobic | 25-Feb | 7-Mar | 10 | 1 | 1 |
| Aerobic | 29-Mar | 5-Apr | 7 | 2 | 6 |
| Aerobic | 29-Mar | 8-Apr | 10 | 2 | 8 |
| Aerobic | 11-Apr | 13-Apr | 2 | 2 | 9 |
| Aerobic | 11-Apr | 15-Apr | 4 | 2 | 5 |
| Aerobic | 5-May | 9-May | 4 | 2 | 5 |
| Aerobic | 5-May | 11-May | 6 | 1 | 4 |
| Aerobic | 3-Oct | 5-Oct | 2 | 2 | 21 |
| Aerobic | 3-Oct | 7-Oct | 4 | 2 | 12 |
| Aerobic | 3-Oct | 11-Oct | 8 | 2 | 11 |
| Aerobic | 29-Sep | 7-Oct | 8 | 2 | 13 |
| Anaerobic | 5-May | 9-May | 4 | 1 | 3 |
| Anaerobic | 5-May | 12-May | 7 | 1 | 5 |
| Anaerobic | 15-Sep | 19-Sep | 4 | 3 | 19 |
| Anaerobic | 15-Sep | 21-Sep | 6 | 2 | 22 |
| Anaerobic | 15-Sep | 22-Sep | 7 | 2 | 20 |
| Anaerobic | 16-Sep | 19-Sep | 3 | 1 | 10 |
| Anaerobic | 16-Sep | 21-Sep | 5 | 2 | 15 |
| Anaerobic | 16-Sep | 22-Sep | 6 | 1 | 8 |
| Anaerobic | 18-Sep | 21-Sep | 3 | 2 | 25 |
| Anaerobic | 18-Sep | 23-Sep | 5 | 2 | 9 |
| Anaerobic | 19-Sep | 23-Sep | 4 | 3 | 31 |
| Anaerobic | 23-Sep | 26-Sep | 3 | 1 | 5 |
